# Why current quantitative serology is not quantitative and how systems immunology could provide solutions

**DOI:** 10.1007/s42977-020-00061-1

**Published:** 2021-02-20

**Authors:** József Prechl

**Affiliations:** 1 R&D Laboratory, Diagnosticum Zrt., Attila út 126, Budapest, 1047 Hungary; 2 MTA-ELTE Immunology Research Group, Eötvös Loránd University, Pázmány P. s. 1/C, Budapest, H-1117 Hungary

**Keywords:** Serology, Antibody, Quantitative, Systems, Immunology

## Abstract

Determination of the presence of antibodies against infectious agents, self-antigens, allogeneic antigens and environmental antigens is the goal of medical serology. Along with the standardization of these tests the community also started to use the expression “quantitative serology,” referring to the fact that arbitrary units are used for the expression of results. In this review I will argue against the use of the term quantitative serology for current tests. Because each test and each antibody isotype determination uses its own references, the term semiquantitative better describes these methods. The introduction of really quantitative serology could both benefit from and drive forward systems immunological approach to immunity.

## A brief history of serology

Serology in the broader sense means the study of reactions of body fluids and primarily that of blood serum. The root word “ser-” has Indo-European origins and refers to something that flows and moves; hence, the watery flowing part of fluids, like the clear yellowish part of blood after clotting, is called serum. In a stricter sense, as it will be used throughout this paper, serology is the study of immunological reactions of serum. As such, the earliest descriptions of serological assays examined the activity of serum against infectious agents, like bacteria. Jules Bordet classified bacteria-killing agents in serum as heat stable and heat labile. The first turned out to be the glycoproteins, and we now call immunoglobulins; the second came to be known as the complement system. With the advance of transplantation immunology and the description of autoimmune reactions we also learned that these reactions could be directed against non-infectious non-self and even self, respectively. Immunoglobulins are also called antibodies, a term coined to reflect the ability of these molecules to counteract foreign entities, called antigens, in the host organism. A serum containing antibodies against a particular target antigen is therefore called an antiserum. Methods based on the measurement of antibody–antigen interactions are called immunoassays in general; however, this term is more often used to describe techniques where the analyte is the antigen and antibodies are used for capture and/or detection. This review discusses technologies for the characterization of antibody reactivity in serum.

A wide range of laboratory methods had been devised to detect and characterize the immunological reactivity of serum; the complete introduction of these is beyond the scope of this article. Instead, we shall discuss one group of methods that is perhaps the most widely used, is applied to all areas mentioned above (diagnosis of infections, characterization of alloreactivity and diagnosis of autoimmunity and allergy) and is often regarded as a quantitative assay. These methods use labeled antigen or antibody and are called enzyme immunoassays or EIA (Van Weemen and Schuurs 1971; Schuurs and van Weemen 1980). The method most often uses a sorbent, i.e., a solid surface, where the targets of antibodies are immobilized. The reaction between the serum and the antigen is followed by the detection of bound antibodies via the label, which can be enzyme, but also fluorophore, biotin or other detectable moieties.

EIA and methods based on the same concept but somewhat different implementations have been used for serological measurements since 1971 (Van Weemen and Schuurs 1971). Introduction of the method into medical diagnostics required the standardization of reagents, protocols and evaluation methods in order to allow inter-laboratory comparisons to be made. In the first decades most laboratories had their own, self-developed in-house assays. With the advent of biochemistry and molecular biology crude antigen extracts had been replaced highly purified or recombinant or synthesized molecules. Dedicated tools and devices had been developed, surface and conjugation chemistries optimized, processes automated. International cooperation resulted in the availability of reference samples to help compare different technologies, results of different laboratories. Recently the concepts of systems serology (Arnold and Chung 2018) and serolomics (Dillner 2005) have been introduced, referring to the complex computational analysis of high-throughput experimental data for the deep analysis of humoral immunity, with the aim of detailed characterization of antibody features associated with function. Huge databases are dedicated to the collection of experimental data on antibody binding to patches of the antigen, called epitope (Mari and Scala 2006; Vita et al 2019).

So have we really mastered serological assays?

## Conceptual shortcomings of current serological tests

Current serological diagnostic tests are developed with basically one goal in mind: identify reaction conditions that provide the best diagnostic accuracy for that particular classification in a single measurement. Has the person been infected by the SARS-2 virus or not? Does the person have pathogenic anti-DNA antibodies or not? To this end, usually the optimal antigenic molecule and antigen immobilization chemistry needs to be identified, so that upon detection of antibodies the reactivity will be characterized by a number, which falls either in the negative or positive clinical diagnostic category. Consequently, optimization is carried out for each antigen and every relevant immunoglobulin isotype independently. As a result, a single, simple, cheap and fast measurement is expected to yield a single number that describes the complex interactions between potentially hundreds of antibodies differing in affinity, fine specificity, isotype and concentration. This is of course impossible, for several reasons.

**Fig. 1:**
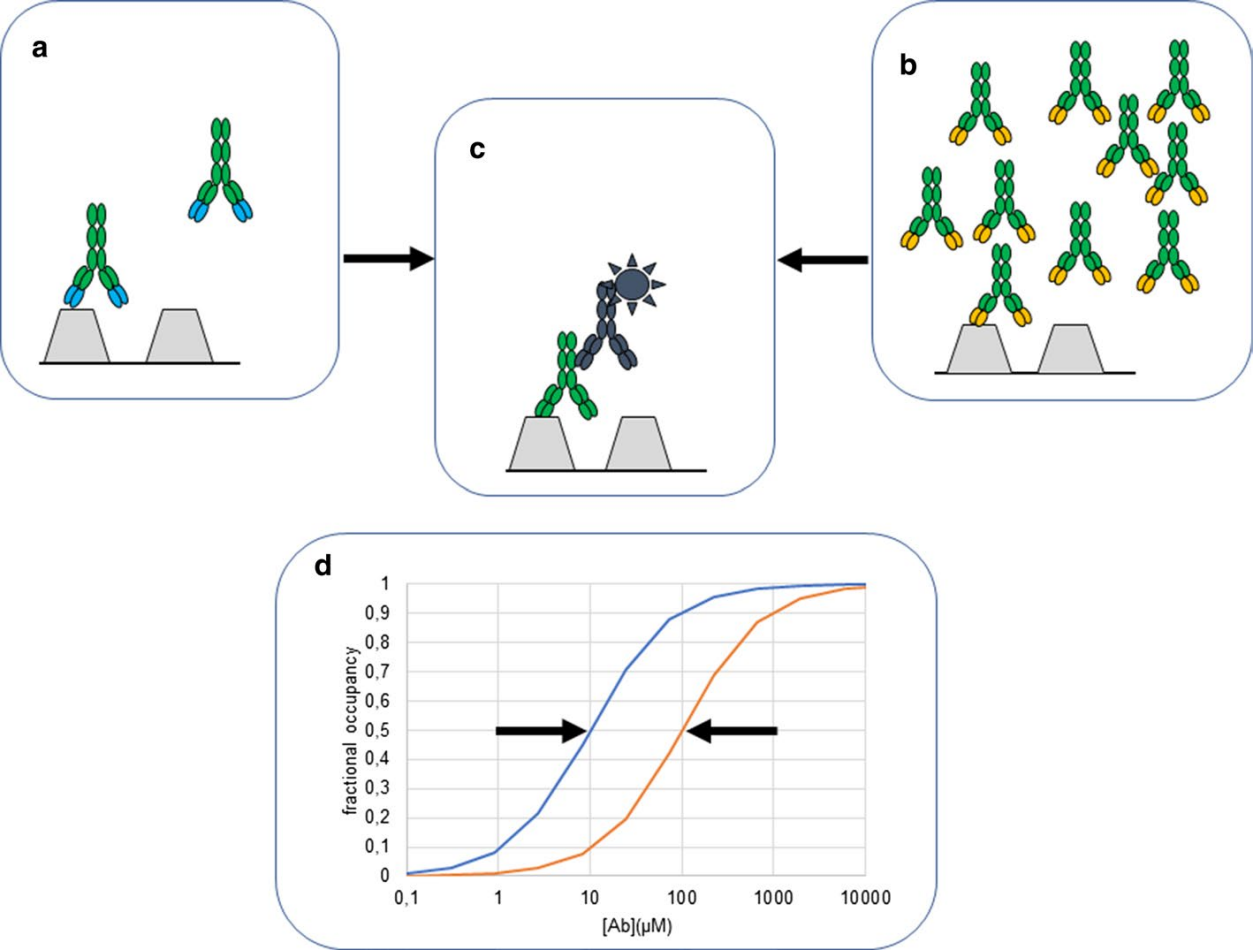
Fractional occupancy as a function of affinity and concentration. Under equilibrium conditions low concentration of high affinity **a** and high concentration of low affinity **b,** antibodies can result in identical occupancy of antigen **c**, expressed as fractional occupancy **d**. Gray polygons represent immobilized antigen molecules, and antibodies are green with their antigen-binding domains color coded according to affinity. Labeled antibody is black and is used for detecting antigen-bound serum antibodies

First, these assays detect the occupancy of the antigen (Fig. 1). A crucial drawback here is that occupancy of the antigen is determined by (at least) two factors: affinity and concentration of antibody. The same occupancy can be achieved by higher concentration of low affinity antibodies and lower concentration of high affinity antibodies. This is an important confounding factor in the interpretation of results, since the readout of the assay is dependent on the occupancy only and we have no a priori knowledge of concentration nor of affinity.

**Fig. 2:**
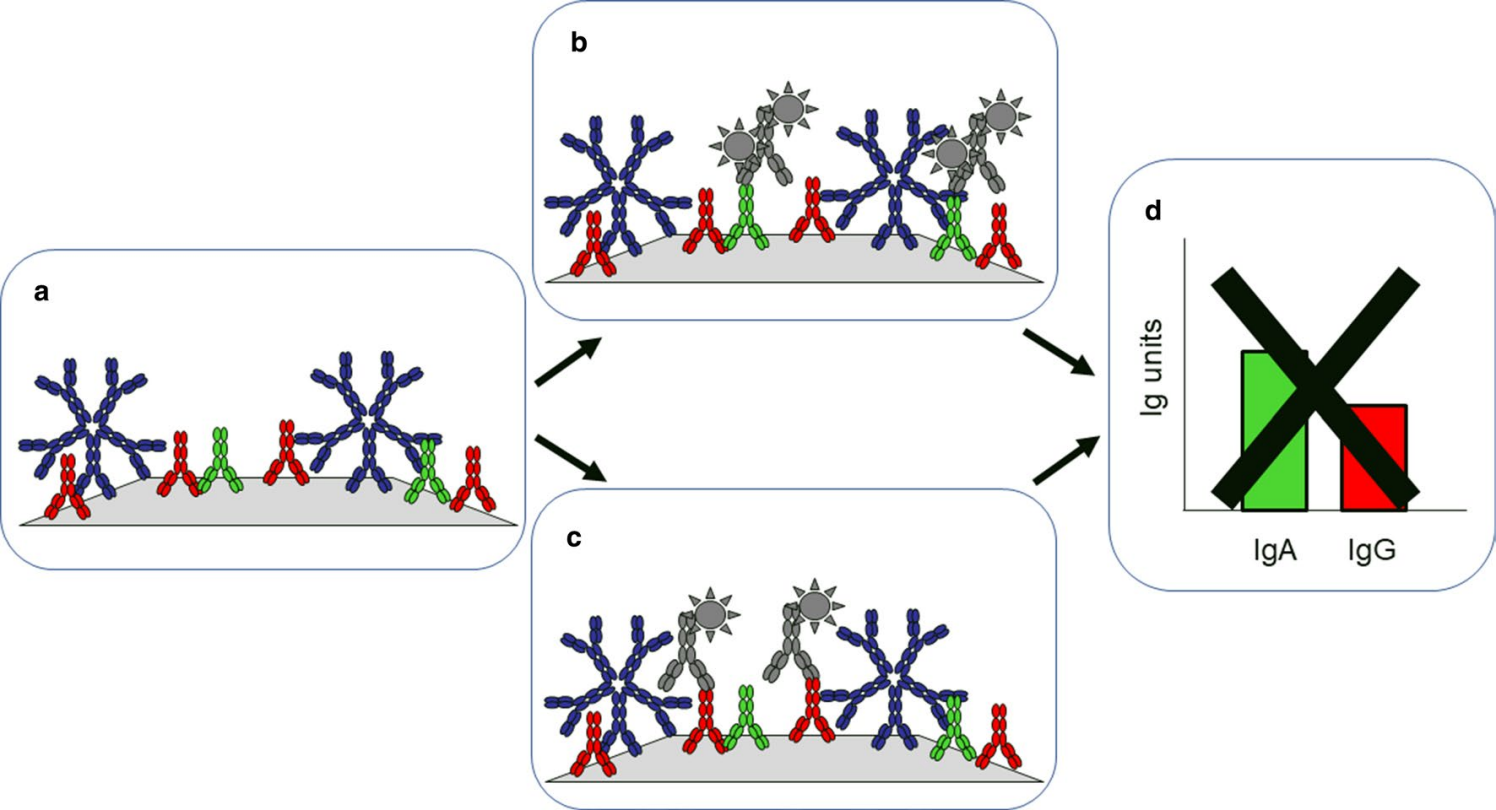
Units of reactivity of distinct isotypes are not comparable. While incubating serum with immobilized antigen, all components of serum are capable of binding the antigen bind and remain bound after washing **a**. Subsequently, selected components (immunoglobulin isotypes) are detected with different reagents (**b** and **c**) and units calculated based on different reference materials. Without the normalization of detection methods, units of distinct isotypes for the same antigen specificity are not comparable **d**. Serum immunoglobulins are shown in blue (IgM), red (IgG) and green (IgA); labeled antibodies are gray

Second, detection of distinct antibody isotypes is not normalized and is therefore not comparable (Fig. 2). Does it matter? We cannot tell, because no systematic inquiry has been made, given the lack of proper technology. We do know that certain isotypes are more relevant for the diagnosis of particular conditions. For example, celiac disease is diagnosed by the presence of anti-transglutaminase antibodies of IgA class in adults, but IgG is also important in children and in IgA deficiency. Immunoglobulin specific antibodies, called rheumatoid factors, are used for the diagnosis of rheumatoid arthritis, but only of the IgM class while it is known that rheumatoid factors of other antibody classes are also present. A better knowledge of the relative timing and extent of isotype switching, generating novel isotypes, would presumably improve our diagnostic and prognostic abilities. In short, if results of serological tests for identical antigen and distinct antibody isotype were quantitatively comparable, it would open up a completely new way of analyzing disease states.

Third, serological test results for distinct antigens are not comparable. For clinical diagnostics it may sound completely irrelevant for a particular diagnostic test to be comparable to another test with a molecularly unrelated target. We tend to regard these tests unrelated, assuming an infinite diversity of potential targets of the immune system. While this potential is indeed virtually infinite when the immune system starts to develop, development itself is about defining a hierarchy of molecular targets classified by the risk it poses to the host. Thus, throughout the life of the host the immune system adjusts affinity against selected antigenic targets. The number of long-lived plasma cells is limited, the number of memory B cells is limited and these limits force a network structure upon the system. In order to promote the experimental mapping of this network, a systems immunological approach does require all results to be comparable. Systematic mapping of the antibody interaction network is expected to reveal relationships and associations in immune reactivity that are currently hidden because of technological limitations. As a timely example, the current controversy over the speed of decay of anti-SARS-CoV-2 antibodies partly derives from the use of different experimental methods and quantitation methodologies (Bölke et al 2020).

## An ideal quantitative serological test

Based on the above-listed concepts, we can formulate the properties of quantitative systems immunological serological tests. Such an ideal serological test should yield results that are

1. Indicative of both affinity and concentration of specific antibody
2. Quantitatively comparable for distinct isotypes against same antigen
3. Quantitatively comparable for distinct antigens
4. With quantitative universal units of measurement
5. Based on molecularly defined antigen targets

With these goals in mind, we shall look at two fundamentally different approaches: one that actually circumvents the fine characterization of antibody binding by examining the biological effects of binding instead, and another one that attempts to deeply characterize antibody binding in its full complexity.

## Quantitation of biological effects

Even though biological responses themselves are more complicated than binding reactions, the readout of a biological response can be simpler than that of antibody binding. The reason for this simplicity is the convergence of antibody effector functions: no matter which antibody isotype, what affinity and concentration, what glycoform initiates the response, the effector function can be essentially the same. Our group has developed two approaches to measure antibody function in an antigen specific manner: complement fixation (Papp et al 2007, 2012a; Prechl et al 2012) and cell activation (Szittner et al 2013). Both of these platforms utilized protein microarray technology, with antigens being printed as arrays of microspots on a solid surface (Prechl et al 2010; Herbáth et al 2014). This methodology inherently satisfies condition 3, comparability of distinct antigens, at least for the antigens on a given microarray, since these are reacted and developed under identical conditions.

**Fig. 3:**
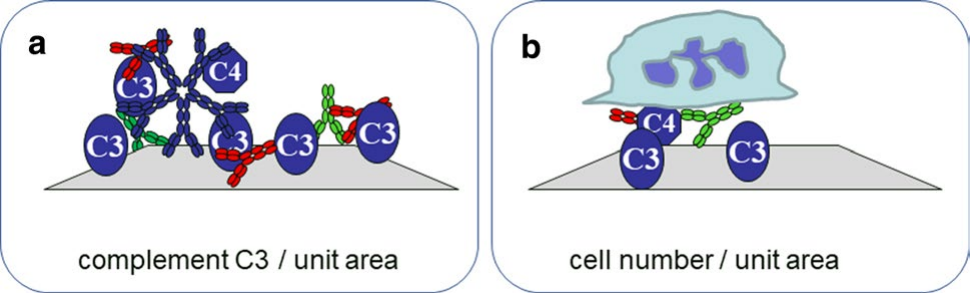
Quantitation of biological responses. Effects of distinct antibody isotypes converge in biological responses, as complement fixation **a** and cell activation **b**, which can be characterized by the density of complement molecules or cells

The complement system is an ancient biological machinery, part of the innate immune system, composed of recognition molecules, serine proteases, their regulatory molecules and receptors. There is a bidirectional interplay between complement and humoral immunity: the nature and extent of complement activation tunes adaptive immunity (Erdei et al 1991, 2003, 2009; Molnár et al 2008a, 2008b; Sándor et al 2009), which in turn exploits effector functions of the complement system (Erdei et al 2016; Lukácsi et al 2017, 2020), both for the better (protection against pathogens) or for the worse (self-destruction in autoimmunity). Antibodies trigger complement activation and this property has long been used in complement fixation assays. By measuring complement fixation, namely complement C3 and C4 deposition on antigen microarrays we were able to quantify this property of antigen-bound antibodies (Papp et al 2008a, 2008b). This, in turn, allowed us to qualify humoral immunological responses and characterize disease (Papp et al 2010, 2012b; Prechl et al 2016). Owing to the fact that the three complement activation pathways meet at complement C3 fragment cleavage, the measurement of C3 deposition can provide a general readout for different activation events. Quantitation of C3 density following the reaction between serum and immobilized antigen is therefore a robust means of assessing serum reactivity against the particular antigen (Fig. 3a).

Receptors of antibodies displayed by various white blood cells contribute to the control of survival and activation of these cells (Sándor et al 1979; Verbeek et al 1997; Nimmerjahn and Ravetch 2007). Fc receptors, which bind the non-variable stalk part of antibodies can efficiently trigger the uptake of antigen, the proliferation of cells, the secretion of cytokines, among other cellular functions (de Taeye et al 2019). Distinct immunoglobulin isotypes possess widely variable effector functions (Vidarsson et al 2014), so the measurement of cellular effector functions reflects the combined, integrated view of all different antigen-bound antibodies via the “eyes” of cells: Fc receptors. By loading white blood cells onto protein microarray chips (Fig. 3b), we were able to identify antigens against which antibodies were present and characterize the cell-activating property of those antibodies in antigen specific manner (Szittner et al 2013, 2016; Kecse-Nagy et al 2016). This approach is particularly exciting when cells from the examined individual are used, since in this case in vivo conditions are simulated in vitro: interactions between own immunoglobulins and cells reveal such subtle differences that are completely concealed by traditional serological assays. These include allotype and glycoform differences of the antibodies (Dekkers et al 2017) and FcR allotype and expression differences on the cells (Li et al 2014). In this sense this is “personal diagnostics” referring to the fact that the same antibodies can induce different biological responses in a different individual (owing to genetic and epigenetic differences in cells) and the same cells can be instructed differently by similar antibodies from different individuals (owing to differences in allotype and glycosylation). Implementation of these tests as point-of-care diagnostics by using microfluidic chips could be a promising step toward quantitative personal serology (Papp et al 2017).

The measurement of biological responses, thus, circumvents the need to address antibody affinity and concentration, isotype distribution, and is amenable to multiplexing and quantification. It is also possible to use universal units as readout: concentration of complement proteins on calibrated chips or number of activated cells on a unit surface (Fig. 3). These technologies are therefore suitable for developing quantitative serological assays. What renders these tests less suitable for standardized, accurate methods is the sensitivity of complement and cells to storage and reaction conditions. A possible solution is to exploit these tests as point-of-care technologies for immediate use of sample and quick results.

It is important to remember that biological responses are often if not always dependent on the spatial density of the triggering molecule. Complement activation is influenced by the proximity of antibody molecules, whereas FcR cross-linking efficiency determines signaling events and activation. Thus, the antigen density employed in the assay, which eventually determines effector molecule density, has to be optimized for the particular application. But this issue takes us back to our original problem of non-comparability. Alternatively, antigen density should be titrated within the assay, providing a distribution of responses which then allows a truly quantitative analysis.

Last but not least, the most direct effect of antibody–antigen interaction, neutralization of the target can be quantitated, again circumventing the need of measuring antibody isotypes individually. These tests can be applied when the molecular interaction partner of the antigen is known and available. The presence of antibodies in the serum that inhibit the interaction can be measured by quantitating the interaction itself rather than antibody binding. Neutralization assays are often used to characterize the efficiency of serum antibody at inhibiting viral infection of cells and thus provide very important biological readout but yet fail to meet our above criteria for an ideal assay.

## Deep analysis of antibody response

**Fig. 4:**
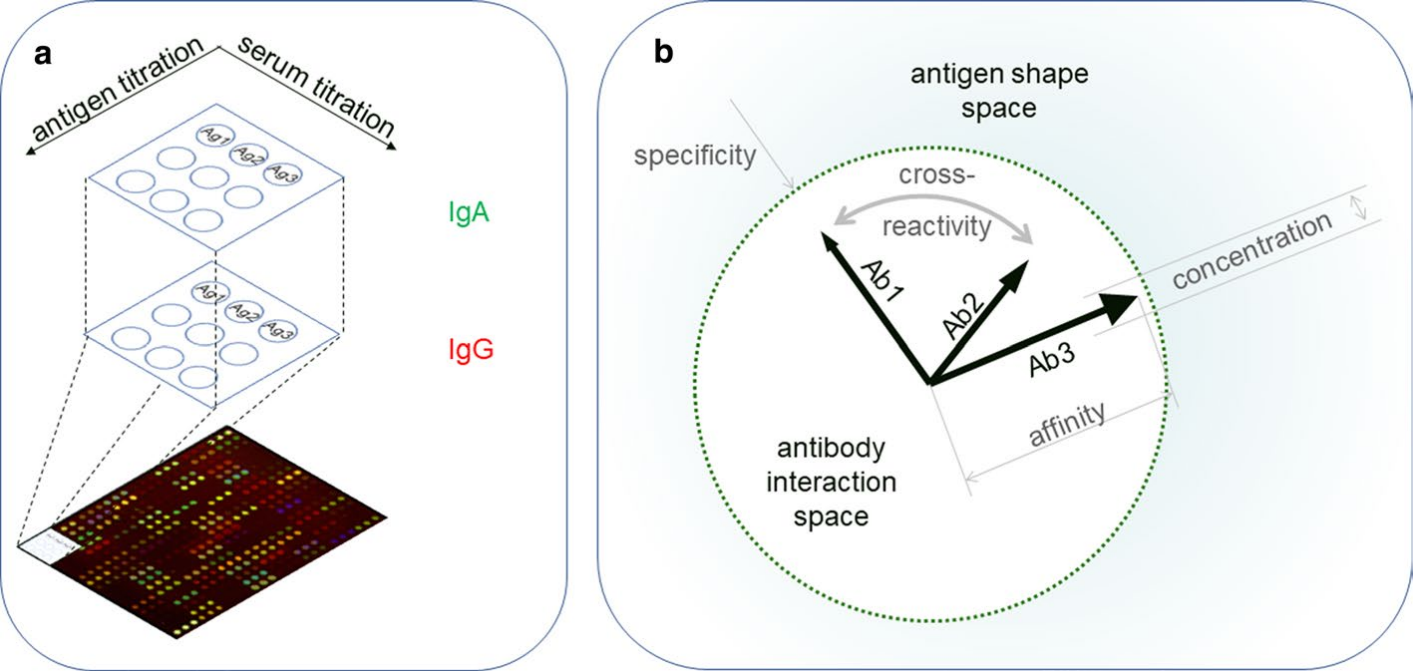
Attributes of serum antibody reactivity measured by an ideal assay. Highly multiplexed and high-throughput measurements can combine the titration of serum and antigen, using multiple antigens and detecting several antibody isotypes in parallel **a**. Systems biology and physics of complex systems can turn quantitative results into a real map of antibody interactions. In a vector space of antibody chemical potential, direction of the vector (arrows) identifies specificity as a point in antigen shape space, magnitude of vector (arrow length) identifies affinity, and angle between vectors defines relative cross-reactivity (bidirectional arrow). Concentration (size of arrowhead) modulates reaction binding energies and absolute cross-reactivity. Three exemplary antibodies (Ab1, Ab2, and Ab3) are shown out of the tens of thousands produced by plasma cells

Based on the above criteria, proper quantitative analysis of serum antibody binding should estimate the concentration and affinity of a given immunoglobulin isotype specific to a given antigen. If molarity is used to express these antibody attributes and a molecularly defined antigen, then all criteria are satisfied. Comprehensive quantitative analysis of serum antibodies, in the sense of measuring binding to lots of antigens, could lead to the mapping of antibody interaction space in antigen shape space (Fig. 4).

It has been appreciated since the earliest studies of serum antibodies (Heidelberger and Kendall 1935; Landsteiner and van der Scheer 1936) that they are heterogeneous with respect to affinity: there is a range of affinities over which the different clones are distributed. Quantitative assessment of affinity therefore requires the characterization of this distribution. This in turn suggests that measurements have to be made over a range of conditions, and antigen titration has indeed been utilized in several different approaches (immunoprecipitation, competition assays, ELISA) to distribution analysis. Currently most widely used kinetics-based methods of affinity measurement (like surface plasmon resonance, quartz crystal microbalance, microscale thermophoresis, etc.) are amenable to automation but are generally not suitable for selective measurement of different antibody isotypes without preanalytical steps. Unfortunately, preanalytical separation not only increases methodological error but also excludes the possibility of measuring the joint effects of all different serum antibodies.

Fluorescent methods have the advantage of selectively detecting the isotypes of choice, ideally without interfering with interactions. If we start with an antigen solution of known concentration and follow the formation of immune complexes or the inhibition of binding of serum antibodies, then we can use the units of concentration for the results. By employing state-of-the-art technologies for liquid transfer and miniaturization of assay, that is, automation, microfluidics and microarrays, it is possible to titrate serum reactivity in a detailed yet affordable and robust manner. Our group is developing an assay that exploits microarrays in a non-traditional manner: instead of making multiplex measurements, in the sense of using multiple different antigens as targets of serum antibodies, we carry out multiple measurements with the same antigen under different conditions. Such an on-chip titration can both simplify and render binding experiments more robust, since all the reactions are carried out in a single microarray chamber (manuscript in preparation).

## Future prospects of quantitative serology

There are two distinct directions of development of serological assays. One is the point-of-care testing for rapid results, with the potential of carrying out the test at home. Tests quantitating the biological effects of antibodies are amenable to miniaturization, microfluidics chip usage and connection to mobile phones. Thus, we can envisage serological chips hooked up with phones that forward results obtained from finger-prick blood to medical centers.

The other direction is the centralized, dedicated laboratory, where accurate, multiplex results can be generated. Such tests are expected to draw a personal immune profile from time to time, controlling vaccine efficacy, allergy treatment efficiency, signs of autoimmunity or tumor development.

Antibody–antigen interactions are interconnected. This phenomenon stems partly from the complexity of antigens: biological molecules are large, and their surface provides multiple binding sites (epitopes) for antibodies. A sufficiently large antigen can bind several different antibodies at a time. On the other hand, antibodies can bind to different antigens, with different affinities: the better the fit, the higher the affinity. These effects are referred to as cross-reactivity. This promiscuity of both binding partners generates relationships, which can be described by networks. Binding networks of antibodies were proposed to be responsible for diversity generation by Niels Jerne (Jerne 1974), and antibody networks based on similar structure and binding patterns had been characterized experimentally (Madi et al 2011; Bransburg-Zabary et al 2013; Miho et al 2019) and theoretically (Manivel et al 2002; Prechl 2017, 2020). Computational power is no longer a limit for the analysis and storage of massive complex data, so we expect that quantitative analysis of binding shall contribute to the generation of individual antibody networks. Characterization of personal antibody networks will help design vaccines, predict, prevent or treat allergy and autoimmunity.

Databases on immunoglobulin sequences are growing continuously, owing to next-generation sequencing being applied to human B cell populations. Once these databases are interlinked to epitope and paratope databases, like iedb.org, it will become possible to identify antigen specific antibodies by mass spectrometry (Bauer and Kuster 2003; Ladwig et al 2017). As mass spectrometry instrumentation is getting cheaper and smaller, the method itself becoming quantitative, it is a potential candidate technology to partially replace current serological tests on the long run (Wang et al 2019). Development of simplex assays with optimal adjustments for gaining biological insight, multiplex systems assays with ability and capacity to generate massive quantitative datasets and theoretical and computational frameworks to integrate all data should proceed in parallel, as these approaches complement each other.

Even though we can sequence a significant part of the human antibody repertoire (Robinson 2015; Kim and Park 2019), identify and quantitate peptides from specific antibodies, we still need advanced serological approaches to obtain a full functional picture of the antibody-OME (Loos et al 2020). In terms of available technology, the stage is set for “next-generation serology.” The important role immunology plays in chronic diseases and in pandemics has turned public attention toward immunology. It is now up to the scientific community to make steps toward the realization and application of quantitative systems serology.
